# Telehealth utilization barriers among Alabama parents of pediatric patients during COVID-19 outbreak

**DOI:** 10.1186/s12913-023-09732-w

**Published:** 2023-06-27

**Authors:** Md Jillur Rahim, Pallavi Ghosh, Anne E. Brisendine, Nianlan Yang, Ryan Roddy, Mia J. Broughton, Alexis Kinzer, Martha Slay Wingate, Bisakha Sen

**Affiliations:** 1 Department of Health Policy & Organization, University of Alabama at Birmingham, 1665 University Blvd, RPHB 330, Birmingham, AL 35233 USA; 2 School of Medicine, University of Alabama at Birmingham, Birmingham, USA; 3 Nutrition Obesity Research Center, University of Alabama at Birmingham, Birmingham, USA

**Keywords:** Telehealth, Telemedicine, Access to Care, Healthcare Utilization, Emergency Department (ED), Children, Pediatric, Parents, Telehealth barriers

## Abstract

**Background:**

Telehealth can improve access to evidence-based care at a lower cost for patients, especially those living in underserved and remote areas. The barriers to the widespread adoption of telehealth have been well documented in the literature. However, the barriers may not be the same for pediatric patients, who must rely on their parents or guardians to make healthcare decisions. This paper presents some of the leading barriers parents or guardians of pediatric patients report in using telehealth to meet their children’s healthcare needs.

**Methods:**

This cross-sectional survey was conducted in a tertiary care pediatric Emergency Department (ED) at a children’s hospital in Alabama between September 2020 to December 2020. The parents or guardians of pediatric patients were asked about their reasons for not using telehealth despite having healthcare needs for their children, whether they canceled or rescheduled healthcare provider visits and facility visits, and whether the child’s health conditions changed over the past three months. Descriptive analyses were conducted that explored the distribution of telehealth use across the variables listed above.

**Results:**

Five hundred ninety-seven parents or guardians of pediatric patients participated in the survey, and 578 answered the question of whether they used telehealth or not over the past three months. Of them, 33.1% used telehealth, 54.3% did not, and 12.6% did not have healthcare needs for their child. The leading reason for not using telehealth was that the doctor or health provider did not give them a telehealth option, the second main reason was that they did not know what telehealth is, and the third leading reason was that the parents did not think telehealth would help meet healthcare needs for their child.

**Conclusions:**

This study highlights the telehealth utilization barriers among underserved pediatric populations, including the need for physicians to proactively offer telehealth options to parents or guardians of pediatric patients. Improving health literacy is of paramount importance, given that a substantial proportion of parents were not familiar with telehealth. Policymakers and healthcare organizations should raise awareness about the benefits of telehealth which can improve healthcare access for underserved pediatric patients.

**Supplementary Information:**

The online version contains supplementary material available at 10.1186/s12913-023-09732-w.

## Background

The outbreak of the COVID-19 pandemic caused significant disruptions to healthcare delivery to children and adults alike in the US and further saw the emergence of telehealth as a critical vehicle for healthcare delivery. Responding to the need to provide access to medical services while keeping COVID-19 transmission risk low and complying with social distancing measures imposed by authorities, the extensive restrictions on telehealth use that had existed prior to the pandemic were significantly reduced [1]. This was facilitated by the Centers for Medicare and Medicaid Services (CMS), extending coverage eligibility for telehealth services, and easing regulatory requirements for telehealth. State authorities and private insurance companies rapidly followed. As a result, telehealth visits quickly grew. For example, less than 1% of outpatient visits were provided through telehealth before the pandemic, 13% between March-August of 2020, and 8% between March and August 2021 occurred via telehealth [1].

In the case of pediatric health care, there has been cautious optimism that telehealth may improve access to care, especially for disadvantaged children, and reduce barriers for parents in terms of taking time off from work and traveling significant distances to access care [1]. At the same time, concerns exist about the barriers to telehealth use, particularly among disadvantaged patients. In an extensive survey of physicians, they reported that the primary obstacles faced by their patients in accessing telehealth were lack of access to technology and broadband, digital literacy, lack of awareness and information about telehealth visits, and beliefs and preferences that in-person visits work better [2]. Results from surveys on telehealth use among adult patients indicate that approximately 23% of patients used telehealth, with higher utilization among publicly insured patients, low-income patients, and patients of color [3, 4]. At the same time, at least one study on publicly insured children's telehealth use indicates that lower-income pediatric patients and patients of color are less likely to use telehealth [5]. Thus, findings about telehealth use and barriers to telehealth utilization among adult patients may not be generalizable to pediatric patients. Improving pediatric healthcare delivery by leveraging telehealth requires understanding the perceptions and barriers to telehealth use among parents and guardians making decisions about seeking healthcare for pediatric patients [6]. However, there is a gap in extant literature that provide insights into the potential reasons among parents for not using telehealth for children, and this study intends to fill that void.

To this end, a survey was administered to parents and guardians of children presenting to the largest pediatric Emergency Department (ED) in Alabama in the fall of 2020 to gather information on their use of telehealth for their child, self-reported experiences, perceptions, and barriers to using telehealth, as well as family socioeconomic status, and experiences of disruptions in healthcare access and utilization in the early months of the pandemic. According to the Commonwealth Fund’s State Health System Performance Scorecard 2022, Alabama ranked 46^th^ in the nation [7]. Further, the state has a median household income of 25% lower than the national average [8]. Thus, this study aims to provide insights into the telehealth utilization barriers for a particularly disadvantaged part of the country and helps inform on inequities that need to be addressed to bring about a true paradigm shift in pediatric healthcare delivery.

## Methods

### Survey design and setting

Data for this study was collected via a survey administered to a convenience sample of parents and guardians of pediatric patients presenting to the tertiary care pediatric emergency department (ED) at Children’s of Alabama (CoA) hospital over twelve weeks from September 18, 2020, to December 11, 2020. Any parent or guardian of medically stable patients presenting to the ED was considered eligible for inclusion. Encounters were excluded if patients were unstable, if parents or guardians appeared intoxicated, if the patient was in Alabama Department of Human Resources (DHR) custody and accompanied on the visit by a DHR officer, or if the parent or guardian did not speak or read English. Data collected from the encounters were limited to the survey completed by parents or guardians; no additional data were abstracted from patients’ medical charts. Families deemed eligible were approached by a research investigator, and the purpose of the study and questionnaire were explained. If they were willing to participate, a research information sheet was provided, and they were given a paper questionnaire for voluntary completion. Because there were no existing validated survey instruments regarding patterns of telehealth use in the early months of the COVID19 pandemic, questions for this survey were formulated in consultation with researchers specializing in pediatric health management during disasters and national emergencies.

### Measures

Telehealth is the exchange of information between healthcare professionals and patients via communication technologies to improve the clinical health status of patients [9, 10]. The term telehealth or telemedicine is generally used as an umbrella term for delivering healthcare services through any information and communication technologies, but the definition of telehealth is much narrower for the scope of this study. Children’s of Alabama Hospital allowed parents to use phone or video visits instead of in-person visits [11].

Parents and guardians were asked about whether they used telehealth services for their children’s health care needs within the past three months, whether their children had any health care needs in that period, and – if they did not use telehealth services despite a health care need – then what their perceived barriers were to telehealth service use. Parents/guardians were also asked about any deterioration in their children’s health condition in the past three months, experience with in-person visits being canceled or re-scheduled, and hesitation about using in-person health care services for their children. Additionally, socio-demographic information, including information on the marital status of the parent/guardian, age of children in the household, family income, and declines in family income in the early months of the COVID19 pandemic, were collected. Due to facility restrictions encountered by the research team at that time, the information on race or insurance status could not be included in the survey. The Institutional Review Board of the University of Alabama at Birmingham approved this study.

### Analysis

Descriptive statistics were calculated for demographic characteristics, healthcare provider visits, healthcare facilities visits, and condition of child health. In addition, a chi-squared (${\chi }^{2}$) test of independence was performed to examine the association between telehealth utilization and individual survey items. Furthermore, the key reasons parents decided not to use telehealth despite having healthcare needs for their children were identified. Finally, the leading reasons for not using telehealth were stratified based on socioeconomic status, rescheduling or cancellation of healthcare provider and facilities visit, and whether the child’s health condition changed over the past three months. Household income was used as a proxy for socioeconomic status. Data were analyzed using STATA 17/SE software package.

## Results

A total of 597 individuals participated in the survey. Five hundred seventy-eight respondents responded to the question, *“Over the past three months, did you use telehealth (that is, talk with a healthcare provider by video conferencing using a smartphone, computer, or tablet) for any of your children’s healthcare needs?”* Of them, 33.1% (*n* = 191) used telehealth services, 54.3% (*n* = 314) did not use telehealth, and 12.6% (*n* = 73) stated the question did not apply to them since was no healthcare need. Among the 191 respondents who reported using telehealth, 45.5% (*n* = 87) said they used telehealth more than once. Nineteen did not answer telehealth utilization questions or provided inconsistent responses and were dropped from our final analysis.

Table 1 presents the demographic characteristics of the survey respondents. Most respondents (58%) were married or living with a partner, 32.9% were single, and 9.1% were separated, divorced, or in other relationships. Most survey respondents had children of elementary school-going age or younger. 37.6% of respondents reported that their household income was less than $40,000, around one-third of respondents had income between $40,000 and $100,00, 10.6% had an income of more than $100,000, and 8.6% were not sure about their annual family income. Individuals with a yearly household income of less than $40,000 had more than 65% of respondents who did not use telehealth for their children. Based on Pearson’s chi-square test statistics and associated *p*-values, we did not find a group association between demographic characteristics (marital status, age of children, and household income) and telehealth utilization.

**Table 1:** Characteristics of survey participants

Variable	Overall n (%)	Did Not Use Telehealth n (%)	Used Telehealth n (%)	*p*-value
Marital Status				0.293
Single	166 (32.9)	114 (68.7)	52 (31.3)	
Living with partner	41 (8.1)	25 (61.0)	16 (39.0)	
Married	252 (49.9)	146 (58.0)	106 (42.0)	
Separated/divorced	38 (7.5)	24 (63.2)	14 (36.8)	
Other	8 (1.6)	5 (62.5)	3 (37.5)	
Age of Children in Household^a^				0.135
Infant	160 (32.0)	106 (66.2)	54 (33.8)	
PreK	154 (30.8)	98 (63.6)	56 (36.4)	
Elementary	255 (51.0)	152 (59.6)	103 (40.4)	
Middle School	170 (34.0)	98 (57.6)	72 (42.4)	
High School	120 (24.0)	71 (59.2)	49 (41.8)	
College/working	38 (7.52)	25 (65.8)	13 (34.2)	
Other	14 (2.8)	11 (78.6)	3 (21.4)	
Household Income				0.081
< 20,000 K	90 (18.1)	59 (65.6)	31 (34.4)	
20- < 40,000 K	147 (29.5)	96 (65.3)	51 (34.7)	
40- < 60,000 K	78 (15.6)	39 (50.0)	39 (50.0)	
60- < 80,000 K	45 (9.0)	29 (64.4)	16 (35.6)	
80- < 100,000 K	43 (8.6)	26 (60.5)	17 (39.5)	
> = 100,000 K	53 (10.6)	29 (54.7)	24 (45.3)	
Not sure	43 (8.6)	33 (76.7)	10 (23.3)	

Total Observation, *N* = 505

The overall column provides a column percentage, and the other two provide a row percentage

^a^Column % total is more than 100% because participants selected more than one response

Table 2 shows telehealth utilization by experiences with, or decisions regarding, healthcare utilization. Two hundred fifty-one parents or guardians reported they had to cancel or reschedule 379 appointments with primary care providers (PCP), dentists, or other physicians or providers (specialist, physical therapist, psychiatrist, etc.), averaging 1.51 rescheduling or cancelations per parent. Of those who had to reschedule or cancel PCP visits, 55.4% used telehealth, compared to 44.6% who did not. Among the parents who rescheduled or canceled dentist visits, 51.9% used telehealth, and 48.1% did not. We observe that 57.8% of those who rescheduled or canceled visits to other providers in the past 90 days used telehealth. There was no group association between rescheduling or canceling visits to the providers and telehealth utilization based on Pearson’s chi-square test statistic and associated *p*-value. 185 (37.1%) respondents said they had planned to take their children to an ED or urgent care over the last three months but changed their minds. Of those who changed their mind about ED/Urgent Care visits, 46.5% used telehealth, compared to 32.9% who did not change their mind about ED/Urgent Care visits. Among all the respondents, 73 parents or guardians initially planned for an outpatient visit but changed their minds. Of these parents, 50.7% did not use telehealth, and 49.3% did. The association between ED/Urgent care/outpatient visit planning and telehealth utilization was statistically significant. Ninety-five respondents (20.1%) said at least one of their children experienced a new health problem or the existing health became severe over the past three months. Of them, 50 parents (52.6%) did not use telehealth, and 45 (47.4%) did. The association between new health problems or worsening of existing health conditions and telehealth utilization was statistically significant.

**Table 2:** Telehealth usage by visits to healthcare providers/ facilities

Variables	Overall n (%)	Did Not Use Telehealth n (%)	Used Telehealth n (%)	*p*-value
Canceled or Rescheduled Visits^a^				0.11
PCP, n (%)	157 (62.6)	70 (45.6)	87 (55.4)	
Dentist, n (%)	158 (63.0)	82 (51.9))	76 (48.1)	
Other, n (%)	64 (25.5)	27 (42.2)	37 (57.8)	
Changed Mind on Visits				
ED/Urgent Care, n (%)				0.003
No	313 (62.9)	210 (67.1)	103 (32.9)	
Yes	185 (37.1)	99 (53.5)	86 (46.5)	
Outpatient, n (%)				0.028
No	408 (84.8)	262 (64.2)	146 (35.8)	
Yes	73 (15.2)	37 (50.7)	36 (49.3)	
Other, n (%)				0.30
No	158 (89.3)	110 (69.6)	48 (30.4)	
Yes	19 (10.7)	11 (57.9)	8 (42.1)	
New /Worsen Existing Health Issues				0.034
Yes	95 (20.1)	50 (52.6)	45 (47.4)	
No	377 (79.9)	243 (64.5)	134 (35.5)	

Total Observation, *N* = 505

The overall column provides a column percentage, and the other two provide a row percentage. Column total percentage will be more than 100% for multiple response questions

^a^Column % total is more than 100% because participants selected more than one response

Table 3 presents the distribution of responses to the question, *“If any of your children had a healthcare need in the past three months, and you did not use telehealth, then what were your reasons for not using telehealth? Please select all that apply.”* The leading reason (82 respondents) for not using telehealth was "The doctor or health provider did not give me the option,” the second leading reason was "I did not know what telehealth is” (79 respondents), and the third leading reason was “I did not think telehealth would be useful in meeting my child’s healthcare needs” (58 respondents). Fifteen respondents said they did not use telehealth as they were unsure whether their insurance would cover telehealth visits, eight reported not having access to the internet or smartphone, and two respondents did not feel comfortable using telehealth. The association between reasons for not using telehealth and telehealth utilization was statistically significant based on Pearson’s chi-square test and associated *p*-value.

**Table 3:** Reasons for not using telehealth

	Overall n (%)	Did Not Use Telehealth n (%)	Used Telehealth n (%)
Reasons			
The healthcare provider did not give the option	109 (40.2)	82 (75.2)	27 (24.8)
I did not know what telehealth is	85 (31.4)	79 (93.0)	6 (7.0)
I did not think telehealth would be useful	84 (31.0)	58 (69.0)	26 (31.0)
I did not know if my insurance would cover	17 (6.3)	15 (88.2)	2 (11.8)
No Device/No Internet	10 (3.7)	8 (80)	2 (20)
Not Comfortable	5 (1.9)	2 (40)	3 (60)

The overall column provides a column percentage and the other two columns present row percentages

### Household income and reasons for not using telehealth

**Fig. 1:**
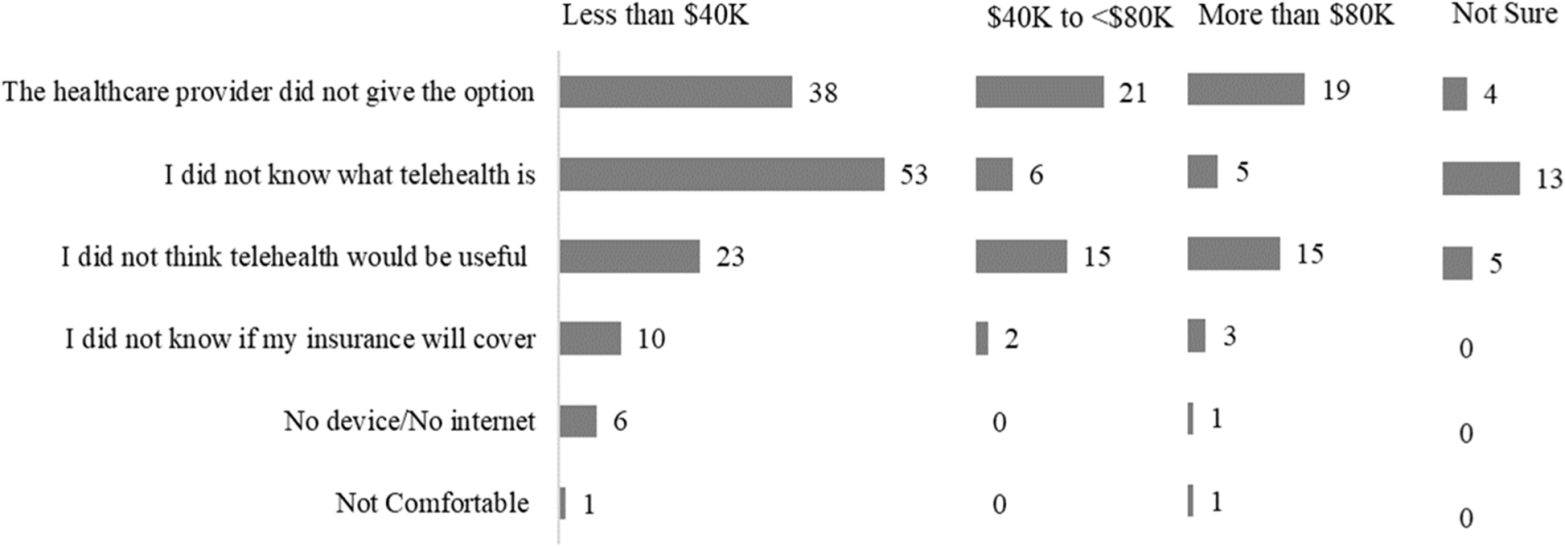
Reasons for not using telehealth by household income of parents/guardians

Figure 1 depicts the reasons for not using telehealth by annual household income ranges. Of those who did not use telehealth despite having healthcare needs for their child, most of them had self-reported family income of less than $40,000. Of the respondents who selected “the healthcare provider/doctor did give the telehealth option, 38 individuals had income less than $40,000, 21 had income between $40,000 and less than $80,000, 19 had income more than $80,000, and four respondents were not sure of their annual household income. Of the individuals who selected “I did not know what telehealth is,” 53 had a family income of less than $40,000. Similarly, for those who selected “I did not think telehealth would be useful in solving healthcare needs of my child,” the highest number of respondents (23) were in the lower income brackets with an annual household income of less than $40,000.

Additional analyses and visuals on telehealth utilization and healthcare provider/facilities visits are included in the Additional file 1.

### Provider visits rescheduling/cancelation and reasons for not using telehealth

Figure A2 provides the breakdown of respondents who did not use telehealth and rescheduled/canceled provider visits. The top reason for canceling PCP and dentist visits was that “the healthcare provider/doctor did not give the option of using telehealth.

### Changing mind about healthcare facility visits and reasons for not using telehealth

Figure A3 presents why parents or guardians initially thought about bringing their children to healthcare facilities (ED, urgent care, outpatient, and others) but changed their minds. The main reason was that “the healthcare provider/doctor did not give the option of using telehealth.”

### Developing new health problems/worsening existing health issues and reasons for not using telehealth

Figure A4 illustrates the reasons for not using telehealth based on the child’s health condition. “I did not think telehealth would be useful in meeting my need” was the key reason for not using telehealth among those whose children had developed new health issues or whose existing health problems worsened in the past three months.

## Discussion

Although there has been an explosion of big data and emphasis on patient-centered health care increased the utilization of digital medicine in recent years [12], telehealth service use was relatively low before the COVID-19 pandemic [1]. The barriers (reimbursement, interstate telemedicine licensing, hospital credentialing) holding back widespread adoption of telehealth were relaxed in the wake of a phenomenal increase in demand for getting health care services using virtual means emanating from the COVID-19 pandemic [13–15].

In this study, 62% of the parents or guardians reported not utilizing telehealth despite having medical needs for their children. The leading reason for not using telehealth among the parents who did not use telehealth is that the doctor or healthcare provider did not give the parents the option of telehealth visits. When we compare the telehealth usage barriers with income, we observe that this is the leading reason among the respondents with a household income of $40,000 or more and the second top reason among people who make less than $40,000 (Fig. 1). This indicates that the physicians need to discuss potential telehealth visits, when appropriate, with parents or guardians of pediatric patients. The second leading reason for not using telehealth was that these parents or guardians did not know what telehealth is, which underscores the importance of health literacy. Of the parents who were not familiar with telehealth conceptually, we observed that two-thirds of these parents or guardians had a family income of less than $40,000. The third top reason for not using telehealth was that the respondents did not think telehealth would help meet their children’s healthcare needs. Of those who selected this reason, we observed that 40% had a family income of less than $40,000. 48% of respondents in our survey had income less than $40,000, but among the non-users of telehealth, 59% had household income less than $40,000. 6.3% of respondents reported not using telehealth because they were unsure whether health insurance would cover telehealth visits.

It is a common perception that the widespread adoption of telehealth will require digital literacy, such as downloading an app, connecting to the internet, or using a particular internet browser. Ortega and colleagues discussed how lack of access to technology could widen disparities among underserved populations with worse health outcomes at baseline [16] and the need for physicians, hospital managers, and policymakers to work together to reduce disparities. To our surprise, only 3.7% of respondents who did not use telehealth reported a lack of internet access or not having a smartphone/laptop as one of the reasons. More than 12% of residents of Alabama lack broadband access compared to 4.4% of residents across the nation [17]. However, it can be conjectured that a driving reason why providers did not give the option of telehealth to patients was either because providers in certain regions lacked technological capacity to offer the service, or they believed that their patients did not have the technical literacy or resources to effectively utilize telehealth even if it were offered. Additionally, it can be conjectured that that the lack of internet and hence to digital information was one underlying reason for many patients not knowing what telehealth was.

In the survey, the participants who canceled or rescheduled medical visits (PCP, dental or non-primary care) reported using telehealth more often than those who did not. Moreover, the parents who thought about bringing their children to healthcare facilities (ED, urgent care, or outpatient visits) but changed their minds used telehealth more than those who did not cancel or reschedule visits. We also found that parents or guardians whose children had either newly developed or worsening health issues were less likely to use telehealth because they did not think telehealth would meet their needs. When a child develops a new health problem or their existing health problem worsens, it is understandable that parents or guardians of pediatric patients would instead prefer an in-person provider or facilities visit. Behavioral changes take time; even though patient satisfaction might be higher with certain telehealth visits [18], in-person visits might be favored by many individuals for testing, medical imaging, dental procedures, etc. In the end, patient and provider preference plays a huge role in the utilization of healthcare services.

Telehealth is likely to remain a significant vehicle for healthcare delivery going forward. However, to improve the telehealth engagement among underserved pediatric population, it is crucial that we address the barriers faced by their parents, guardians, and caregivers. One strategy would be to increases awareness and education about the availability and usefulness of telehealth through targeted education campaigns and outreach activities. As some parents doubt the effectiveness of telehealth in meeting healthcare needs for their children, telehealth interventions should be designed to provide high-quality care and be evaluated to demonstrate their effectiveness [19]. Pediatricians and healthcare providers can play a pivotal role in raising awareness about telehealth by offering telehealth options and providing support to the families [20]. To increases providers’ buy-in, there need to be assurances of reimbursement for telehealth services, so that they will be willing to make necessary investments in technology and patient education [21].

Like most observational studies, there are several limitations to this study. First, the survey design and data collection were influenced by the rapidly changing environment during different waves of the COVID-19 outbreak. We would like to use a validated survey instrument and include additional variables such as modalities of telehealth visits, residence distance from the hospital, type of insurance coverage, and other covariates in the future to shed more light on telehealth barriers in the Deep South region. Second, caution must be exercised when generalizing the study findings, since this was based on a convenience sample of pediatric patients who continued to present to an ED of a children’s hospital during the COVID19 pandemic, and this choice might be influenced by the family’s socio-economic status, as well as attitudes and beliefs about the risk of COVID19 infections [22]. Third, we used income and marital status as a proxy for socioeconomic status, but could not include race-ethnicity information, thus our ability to fully investigate disparities in telehealth utilization is limited. Finally, this study excluded non-English speakers, who may have lower rates of telehealth utilization compared to the overall population and may face barriers that are unique to them [5]. Hence, future studies on barriers to telehealth utilization in this Deep South region should include a more diverse population and include analysis on racial disparities.

## Conclusion

This study can help clinicians and researchers better serve patients by illuminating barriers to telehealth use. Many of these barriers are quickly addressed by strategies as simple as routinely asking patients if they want to see the doctor via telehealth or face-to-face. In addition, policymakers must prioritize health literacy, including easy access to information about whether telehealth visits are covered by health insurance. This will help ensure that patients can access care when needed, with full information on options about the type of visit, and are aware of the potential cost and benefit of their health-related decisions. The findings from this study can be used to design interventions in the post-pandemic era to increase access to telehealth services for underserved pediatric population.

## Supplementary Information

**Additional file 1.**

## Data Availability

The data, codes, and other materials will be available upon request.

## Abbreviations

CMS: Centers for Medicare and Medicaid Services
ED: Emergency Department
CoA: Children’s of Alabama Hospital
PCP: Primary Care Provider
