# Supplementary material for: Telehealth utilization barriers among Alabama parents of pediatric patients during COVID-19 outbreak
Source: BMC Health Serv Res. 2023 Jun 27;23:693. doi: 10.1186/s12913-023-09732-w (PMC10294506; doi:10.1186/s12913-023-09732-w)
Supplement: Supplementary file 1 — Additional file 1. [file 12913_2023_9732_MOESM1_ESM.docx]

**APPENDIX**

**Figure A1: Reasons for not Using Telehealth**


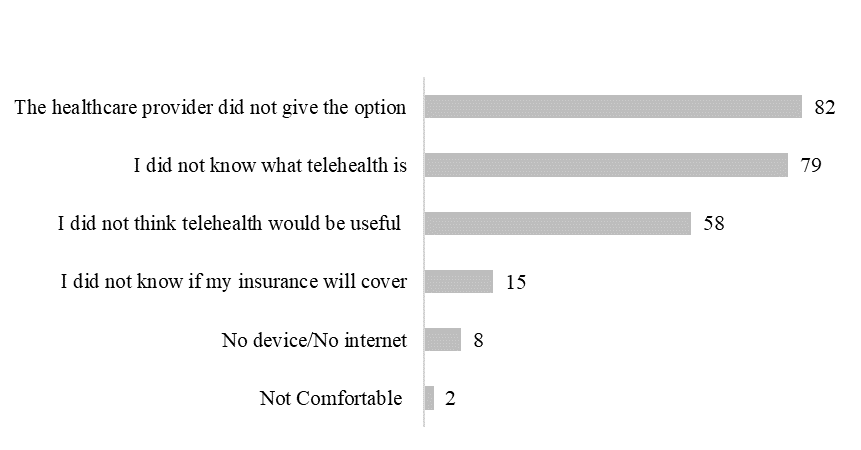


**Figure A2: Reasons for not Using Telehealth by Provider Visits Rescheduling/Cancelation**


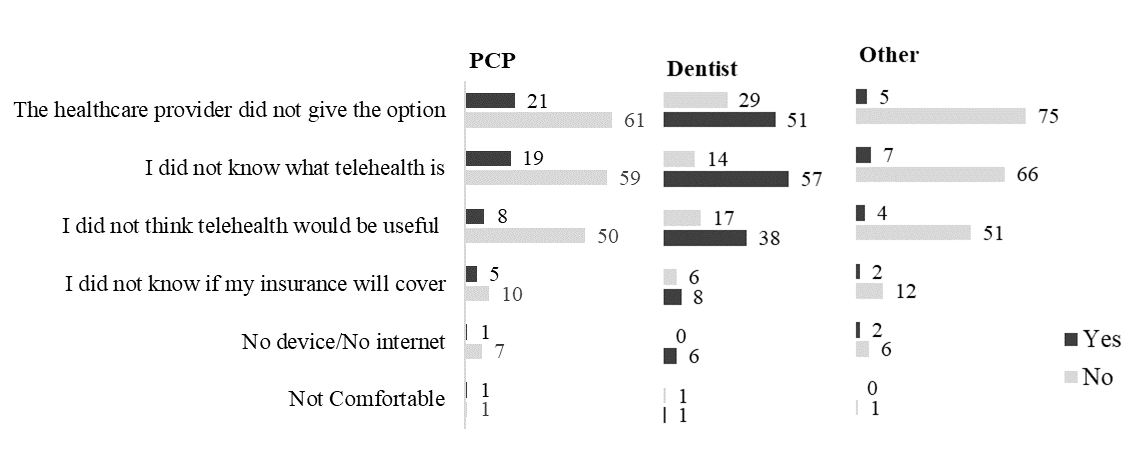


**Figure A3: Reasons for not Using Telehealth due to Change of Mind on Healthcare Facilities Visi****t
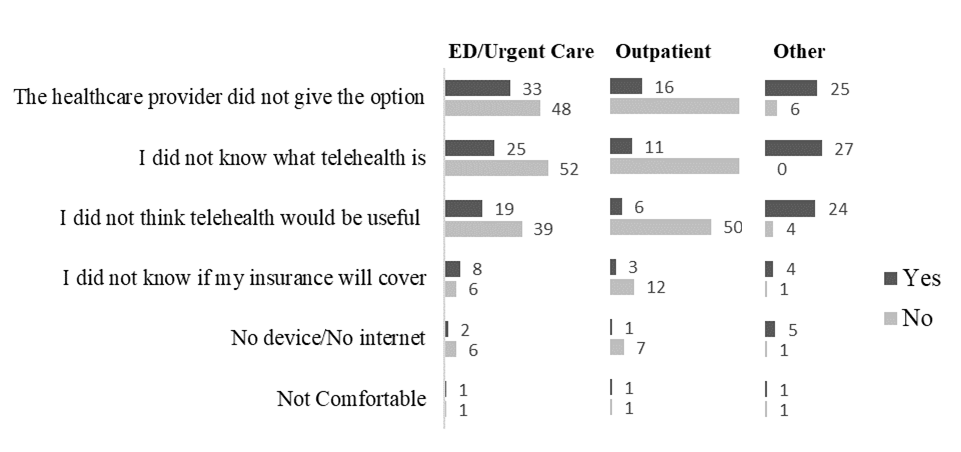
**

**Figure A4: Reasons for not Using Telehealth by Worsening Existing Health Problem(s)/Developing New Health Problems**


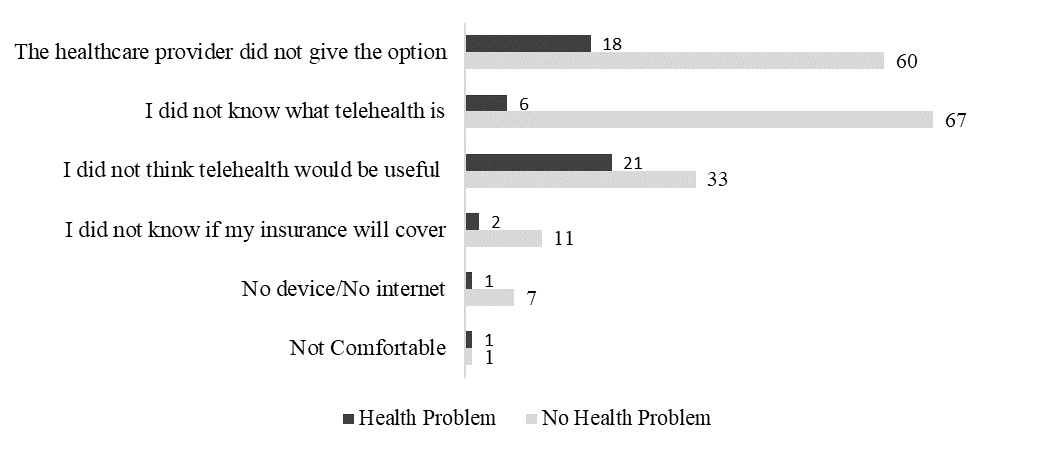


**SURVEY QUESTIONNAIRE**

1. What is your marital status?

- Single 1
- Living with Partner 2
- Married 3
- Separated/Divorced 4
- Other 5

1. Do you have children in your household who are in following categories? Check all that apply
   - Infants 1
   - Preschool 2
   - Elementary School 3
   - Middle School 4
   - High School 5
   - College/Working 6
   - Other (Please Specify) 7 (write in answer too)
2. Please select which category best fits your family income

- <20,000K 1
- 20-<40,000 2
- 40-<60,000 3
- 60-<80,000 4
- 80-<100,000 5
- More than 100,000 6
- Not Sure or do not wish to disclose 7

1. Have you, or your spouse/partner, seen a drop in your income because of the recent shut-downs?

- Yes 1
- No 2
- Not Sure or do not wish to disclose 3

1. Over the last 30 days, how often have you been bothered by the following problems?

|  | Not at all | Just a  few days | Several  days | More than  half the days | Nearly  every day |
| --- | --- | --- | --- | --- | --- |
| Feeling nervous, anxious or on edge | 1 | 2 | 3 | 4 | 5 |
| Not being able to stop or control worrying | 1 | 2 | 3 | 4 | 5 |
| Little interest or pleasure in doing things | 1 | 2 | 3 | 4 | 5 |
| Feeling down, depressed, or hopeless | 1 | 2 | 3 | 4 | 5 |
| Feeling bored, restless, ‘cooped up’ | 1 | 2 | 3 | 4 | 5 |
| Feeling really angry and frustrated | 1 | 2 | 3 | 4 | 5 |

THE NEXT QUESTIONS ASK ABOUT HEALTHCARE NEEDS OF YOU CHILD/CHILDREN DURING THE COVID-19 SHUTDOWN

1. Who would you say makes decisions about healthcare needs about children in your household (like deciding of they need to see a doctor)?

- I make all/most of the decisions 1
- I make all/most of the decisions together with my spouse. 2
- I make some of the decisions. 3
- I do not make the decisions. 4

1. Over the past three months, did you have to cancel/re-schedule any of your children’s visit with any of the following healthcare providers? Please select all that apply. (PUT THREE OPTIONS FOR EACH)

|  | Yes, more  than one visit | Yes, one visit | No | Not  applicable |
| --- | --- | --- | --- | --- |
| Primary Care Provider/Pediatrician  Mental Health Provider (Counselor) | 1 | 2 | 3 | 4 |
| Mental Health Provider (Psychiatrist) | 1 | 2 | 3 | 4 |
| Dentist | 1 | 2 | 3 | 4 |
| Occupational/Speech/Physical Therapist | 1 | 2 | 3 | 4 |
| Specialist (e.g. Cardiology, Pulmonology, Neurology) | 1 | 2 | 3 | 4 |
| Other | 1 | 2 | 3 | 4 |

1. Over the past three months, did you think about, and then change your mind, about taking any of your children to the following?

|  | Yes, more  than once | Yes, once | No |
| --- | --- | --- | --- |
| Emergency Department Visits | 1 | 2 | 3 |
| Urgent Care Visits | 1 | 2 | 3 |
| Outpatient Visits | 1 | 2 | 3 |
| Other | 1 | 2 | 3 |

1. Over the past 3 months, did any of your children developed a new health problem, or have an existing health problem become more severe?

- Yes. 1
- No. 2
- Not Sure. 3

1. Over the past 3 months, did you use telehealth (that is, talk with healthcare provider by video conferencing using smartphone, computer or tablet) for any of your children’s healthcare needs?

- Yes, more than once. 1
- Yes, once. 2
- No. 3
- Not applicable, my children had no healthcare needs in past 3 months. 4
- Not applicable, I took them for in-person visits when needed. 5

1. If any of your children had a healthcare need in the past 3 months, and you did not use telehealth, then what were your reasons for not using telehealth? Please select all that apply.

- I did not know what telehealth is. 1
- The doctor/ healthcare provider did not give me the option to use telehealth. 2
- I did not know if my insurance will cover telehealth 3
- I did not have a computer or smartphones or tablet other devices to use telehealth. 4
- I did not have access to internet or wi-fi to use telehealth. 5
- I did not want to download new apps or software to use telehealth. 6
- I did not feel comfortable or safe using telehealth (e.g. privacy concerns). 7
- I did not think telehealth would be useful in meeting my child’s healthcare needs. 8
